# Unified Interpretations of Two Kinds of Needle-Shaped Precipitates Using Transmission Electron Microscopy and Small-Angle Neutron Scattering in Aged Al–Mg_2_Si(-Cu) Alloys

**DOI:** 10.3390/nano14020176

**Published:** 2024-01-12

**Authors:** Amalina Aina Kaharudin, Masato Ohnuma, Seungwon Lee, Taiki Tsuchiya, Yuuki Asada, Ken-ichi Ikeda, Kazuki Ohishi, Jun-ichi Suzuki, Kenji Matsuda, Tomoyuki Homma

**Affiliations:** 1 Department of Science of Technology Innovation, Nagaoka University of Technology, Nagaoka 940-2188, Japan; 2 Division of Applied Quantum Science and Engineering, Faculty of Engineering, Hokkaido University, Sapporo 060-8628, Japan; 3 Graduate School of Science and Engineering for Research, University of Toyama, Toyama 930-8555, Japan; 4 Department of Mechanical Engineering, Nagaoka University of Technology, Nagaoka 940-2188, Japan; 5 Division of Materials Science and Engineering, Faculty of Engineering, Hokkaido University, Sapporo 060-8628, Japan; 6 Neutron Science and Technology Center, Comprehensive Research Organization for Science and Society (CROSS), Tokai 319-1106, Japan

**Keywords:** pseudo-binary Al–Mg_2_Si alloy, Cu addition, MgSiMg second cluster, transmission electron microscopy, small-angle neutron scattering

## Abstract

This study investigates the nanostructural properties of pseudo-binary Al–1.0Mg_2_Si (mass%) alloys with and without 0.5Cu using transmission electron microscopy (TEM) and small-angle neutron scattering (SANS). The TEM results show that both alloys exhibit extra electron diffraction spots related to MgSiMg second clusters at peak-aged conditions. High-resolution TEM images have revealed that the second cluster exists as a needle-shaped precipitate that is shorter and thicker than the *β*″ phase. We found that the second cluster, which we referred to as the R phase in this paper, is more likely to form partially along the longitudinal axis of a random-type precipitate. Thus, the atomic arrangement in the random-type precipitate is not completely random. SANS is used to quantify the size and volume fraction of the observed needle-shaped precipitates since the R phase is difficult to observe with TEM. The R phase forms even in the Cu-free alloy, but the volume fraction is low, and the growth and formation are retarded near the peak-aged conditions. Undoubtedly, the Cu addition has the effect of stabilizing the growth of the R phase and also promoting its formation. Therefore, the R phase also contributes to the increase in hardness at both under- and peak-aged conditions in the Cu-containing alloy in addition to the strengthening *β*″ phases.

## 1. Introduction

The pseudo-binary Al–Mg_2_Si alloy is one of the most widely used age-hardenable 6000 series Al alloys due to its high specific strength, corrosion resistance, formability, and relatively low cost [1]. The general precipitation sequence of the pseudo-binary Al–Mg_2_Si alloy is given as follows [2,3,4]:

Supersaturated solid solution (SSSS) → Mg–Si and Si-rich clusters → monolayer Guinier–Preston (GP) zone → multilayer GP zone → random-type precipitates → *β*″ → *β*′ → *β*.

SSSS is decomposed into clustering and co-clustering of Mg and Si atoms prior to the formation of the GP zones. Early research on those formations of clusters was proposed by Pashley et al. regarding a two-step aging behavior [5,6]. Edwards et al. reported on the independent Mg and Si cluster formations, followed by the dissolution of the Mg cluster and formations of the Mg–Si cluster during aging at 343 K using differential scanning calorimetry and atom probe field ion microscopy [7]. The Mg–Si clusters formed with pre-aging at 343 K increase the density of the strengthening phase of *β*″, whose unit cell has a monoclinic structure, space group C2/m with a stoichiometric composition of Mg_5_Al_2_Si_4_, and cell parameters of a = 1.516 nm, b = 0.405 nm, c = 0.674 nm, and *β* = 105.3° [3]. On the other hand, the Si-rich clusters that form when the alloy is pre-aged below 343 K or near room temperature are not related to the nucleation of *β*″, since they have a significantly strong Si–Si covalent bond [8]. Even though the Mg and Si contents in the Mg–Si and Si-rich clusters are always emphasized, they are actually composed of all of the elements in the alloy, which are Al, Mg, Si, and also vacancies [9]. The transition from these early two clusters into GP zones was proposed by Huppert et al., where the clusters partially dissolved to allow the Al atoms to diffuse from the clusters to the matrix, and then the GP zones composed of Mg and Si atoms together with the vacancies were finally formed [9].

The structure of the GP zone in a pseudo-binary Al–Mg_2_Sialloy was first proposed by Matsuda et al. in 1998. It is a fine plate that is 1 atomic layer thick, 2.5 nm wide, and less than 30 nm long [3]. The GP zone possesses a periodic arrangement of Mg and Si atoms, having a spacing of 0.405 nm along the <001>_α_ axis, and its presence is confirmed by using high-resolution transmission electron microscopy (HRTEM) [3]. One GP zone is called a monolayer GP zone, but when the monolayer GP zones aggregate with each other, and sometimes with locally Al-rich areas, they are referred to as a multilayer GP zone [3]. The GP zones in pseudo-binary Al–Mg_2_Si alloys directly transform into the *β*″ phase [3], unlike in Si-rich alloys, where initial *β*″ and pre-*β*″ phases form before transforming into the *β*″ phases [10,11,12].

Marioara et al. reported that random-type precipitates or disordered phases are mainly observed when the Mg and Si mol% contents in the alloy composition are higher or lower than Mg/Si:5/6 (=0.83) [13]. Our case is Mg/Si = 1.94 in both the Cu-free and the Cu-containing alloys, as will be discussed later. When there is Mg or Si enrichment around the GP zones, it triggers the disordered arrangement of the *β*″ phases and forms the random-type precipitates [13]. In addition, it is stated that the random-type precipitates also had a needle-shaped morphology like the *β*″ phases, even though the morphology from their longitudinal axis was only confirmed through bright field images and not the HRTEM ones [13].

The unique precipitation behavior in 6000 series alloys has prompted extensive research into optimizing their mechanical properties, such as with a trace addition of Cu. The addition of Cu increases the peak hardness of the alloys, as it helps with the refinement of the precipitates and alters the precipitation sequence. The Cu atoms tend to segregate on the interfaces of the needle-shaped precipitates, like GP zones and *β*″ phases, with the matrix to relax the lattice distortions caused by the structural differences [14], preventing the growth of the diameter of the precipitates but promoting their length extensions through the removal of some Cu atoms from their edges [15]. The general precipitation sequence in the Cu-containing alloys has been summarized in the literature [16,17,18,19,20]. However, the precipitation sequence does not change significantly with a low Cu addition in the pseudo-binary Al–Mg_2_Si alloy, where at least as low as 0.3 mass% has been confirmed [19]. Moreover, Cu-containing precipitates mostly influence the over-aged conditions [21,22,23,24].

In 2017, Matsuda et al. discovered the presence of extra diffraction spots in an age-hardenable Al–1.0Mg_2_Si (mass%) alloy with a 0.5 mass% Cu addition (Cu-containing alloy) that appear in their selected-area electron diffraction (SAED) patterns obtained from a <100>_α_ zone axis [25]. These extra diffraction spots could be observed in under- and peak-aged conditions measured at 473 K. However, the extra diffraction spots could not be confirmed when Cu was not added to the alloy (Cu-free alloy). From the SAED simulation results, they proposed the initial clusters in the Cu-free and Cu-containing alloys, which were based on the structure of the *β*″ phase, to be MgSiMg, CuMgSi, or AlCuMg; however, these initial clusters did not contribute to the extra diffraction spots in the SAED patterns. The second clusters, which consisted of three initial clusters, including an anti-phase boundary with a short range order within the unit cell, were responsible for the extra diffraction spots [25], and they were confirmed not only in the Al–Mg–Si–Cu alloy, but also in the Al–Zn–Mg–Cu and Al–Mg–Ge–Cu alloys through the SAED patterns [25]. Nevertheless, the formation behavior of the second clusters and their relations with other solute clusters in the pseudo-binary Al–Mg_2_Si alloy were still unclear, as the cluster formations and phase transformations at the initial stage of aging were hard to observe with transmission electron microscopy (TEM). 

Small-angle neutron scattering (SANS) is a useful technique to obtain the size and total amount of clusters and precipitates quantitatively. Since the evaluated volume is much larger than those of the TEM and atom probe techniques, more accurate representative and statistical values can be obtained as an average value of the evaluated volume. Compared to small-angle X-ray scattering (SAXS), SANS has a big advantage when detecting clusters and precipitates in the 6000 series of Al alloys because of their higher contrasts in the scattering length density than those of SAXS. This is due to the difference in the scattering lengths for Al, Mg, and Si, which are 0.35, 0.52 and 0.42 × 10^−12^ cm, respectively, while X-ray is about same for these elements [26]. There are a few studies on ex situ SANS measurements on polycrystalline samples of the 6000 series alloys that investigate the effects of time, temperature, or heat-treatment processes on the *β*″ phase sizes and density for optimum hardness [26,27,28,29]. An in situ SANS measurement at 453 K was also conducted on a monocrystalline sample of a 6000 series alloy to monitor the growth of the *β*″ phase independently along with the number density [30].

## 2. Materials and Methods

Table 1 shows the chemical compositions of the Cu-free and Cu-containing alloys used in this study in mass% and mol%. The alloys were fabricated by casting in steel molds. They were then homogenized at 813 K for 36,000 s (10 h) in atmospheric conditions before hot and cold rolling. The rolled materials had a thickness of 1 mm. After the cold rolling, they were solution-treated at 848 K for 3600 s (1 h) in atmospheric conditions and quenched into chilled water kept at 273 K. The as-quenched (AQ) sheets were aged at 473 K in an oil bath and quenched again into chilled water kept at 273 K. 

Vickers hardness (HV) tests were conducted using Matsuzawa Seiki MICRO-SA (Akita, Japan) with a 5 N load and 15 s holding time. Sample observations were conducted using TEM (JEOL: JEM-2100F, JEOL Ltd., Tokyo, Japan) operated at 200 kV. TEM specimens were prepared by mechanical polishing and using a precision ion polishing system (Gatan: Model 691, Pleasanton, CA, USA) with the use of liquid nitrogen. 

The time-of-flight SANS experiments were conducted using the small- and wide-angle neutron scattering instrument, so-called TAIKAN (BL15) (Tokai, Japan), installed in the Materials and Life Science Experimental Facility (MLF) at the Japan Proton Accelerator Research Complex (J-PARC), Ibaraki, Japan [31]. The detectors of TAIKAN were composed of four detector banks of small-, middle-, high-angle, and backward detector banks. A combination of these detectors, which could cover a wide solid angle and broad range of wavelengths λ (0.7 ≤ λ ≤ 7.8 Å), realized a wide q-range (0.007 to 17 Å^−1^). The samples for the SANS measurements were prepared by cutting the rolled material into a 10 × 10 mm^2^ area. Eight samples with the same area were prepared for each of the following four conditions: AQ, aging at 240 s, 6000 s, and peak-aged conditions (18,000 s for the Cu-free alloy and 60,000 s for the Cu-containing alloy). During the measurement, the 8 samples were stacked together with pure Al foils, making the measured thickness for each condition 8 mm (the thickness of a sample was 1 mm), and the sample holders, with an 8-mm-diameter mask made by Cd, were used to fix the sample positions.

## 3. Results and Discussion

### 3.1. Age-Hardening Curves

**Figure 1:**
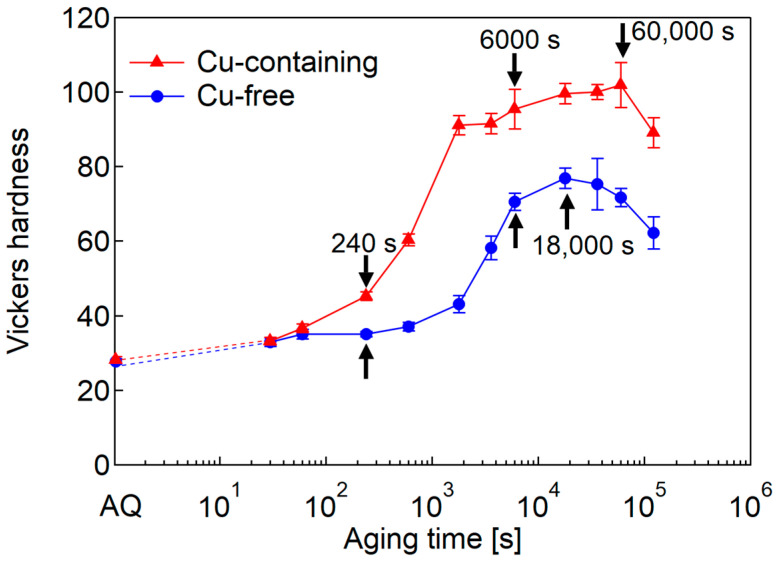
Vickers hardness of Cu-free and Cu-containing alloys at 473-K aging. The black arrows indicate the aging times for the following TEM observations.

Figure 1 shows the age-hardening curves of both of the alloys aged at 473 K. In the AQ state, the Cu-containing alloy shows slightly higher hardness than the Cu-free alloy. In the early stage of the hardening at 240 s, the hardness increase is confirmed for the Cu-containing alloy, though that of the Cu-free alloy does not increase. Then, the hardness in both of the alloys rapidly increases. The peak hardness of the Cu-containing alloy is higher than that of the Cu-free alloy because the Cu addition promotes precipitation refinements with a larger volume fraction, thus, creating more obstacles to hinder the dislocation motion [32]. The black arrows indicate the aging times of the specimens observed by TEM.

### 3.2. TEM Observations

**Figure 2:**
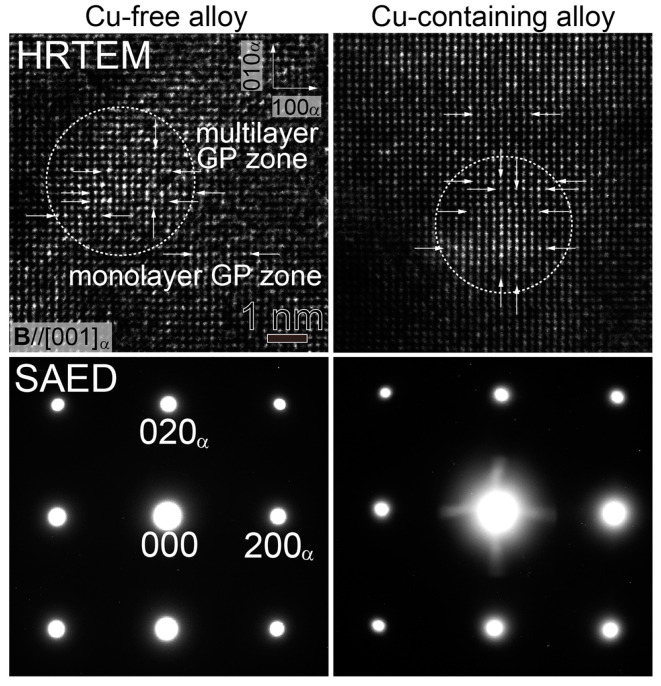
HRTEM images of monolayer and multilayer GP zones, which are indicated with white arrows and a dotted circle in the Cu-free and Cu-containing alloys at 240 s, together with the corresponding SAED patterns.

Figure 2 shows the HRTEM images and corresponding SAED patterns of both of the alloys under-aged at 240 s with monolayer and multilayer GP zones, which are indicated by the white arrows and a dotted circle. The incident beam direction (**B**) is parallel to [001]_α_. The monolayer GP zones can be seen as repetitive bright and dark contrasts, which come from segregations of Mg and Si atoms along <001>_α_, if we accept Matsuda and his coworkers’ results [3]. The multilayer GP zones are determined when there are a few layers of the monolayer GP zone close together, and sometimes with some Al-rich areas in between. There are no extra diffraction spots or streaks in the SAED. The increase in hardness at 240 s in the Cu-containing alloy is due to the high density of the monolayer and multilayer GP zones, where the formation rate is increased by the trace amount of Cu. Although the Cu-free alloy does not show a drastic increase in hardness at 240 s (35.1 HV), the hardness is still higher than that observed at the AQ state (27.7 HV), and monolayer and multilayer GP zones are also observed in this condition. Other precipitates are not yet observed at this condition in both alloys.

**Figure 3:**
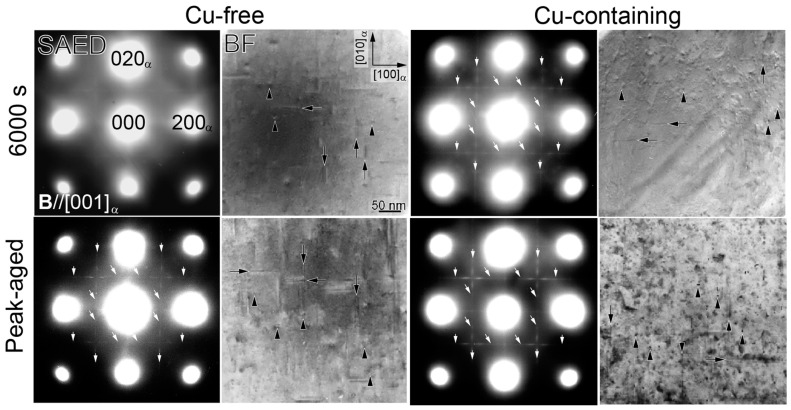
SAED patterns and BF images of Cu-free and Cu-containing alloys at 6000 s and peak-aged conditions. The peak-aged times are 18,000 and 60,000 s, respectively. The incident beam direction is parallel to [001]_α_. The white arrows in the SAED indicate contrast of extra diffraction spots from second clusters, and the black arrows and arrowheads in the BF indicate needle-shaped precipitates and their cross-sectional parts, respectively.

**Figure 4:**
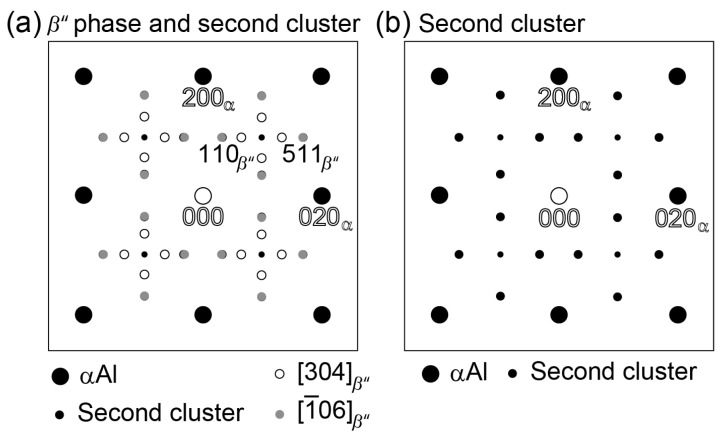
Key diagrams of diffractions of (**a**) needle-shaped [304]_*β*″_, [$\bar{1}$06]_*β*″_, and MgSiMg second clusters and (**b**) MgSiMg second clusters only, obtained when the incident beam is parallel to [001]_α_.

Figure 3 shows the SAED patterns and bright field (BF) images of the Cu-free and Cu-containing alloys at 6000 s and the peak-aged conditions. The times for the peak-aged conditions are 18,000 and 60,000 s, respectively. In the BF images, the black arrows indicate needle-shaped precipitates, which concern either the GP zones or the *β*″ phases, while the black arrowheads indicate fine precipitates that could concern the cross-sectional part of the needle-shaped precipitates. It is known that both the cross-sectional and the longitudinal parts of needle-shaped precipitates can be observed from {001}_α_ [33]. The white arrows in the SAED patterns indicate the contrasts of extra diffraction spots similar to the second clusters reported by Matsuda et al. [25] (Figure 4). In the Cu-free alloy aged at 6000 s, some fine precipitates can be observed in the BF image, while the SAED pattern shows faint streaks that probably correspond to the earlier stages of [304]*_β_*_″_ and [$\bar{1}$06]*_β_*_″_, but no extra diffraction spots. This could be due to the short aging time, the slow nucleation of the second clusters in the absence of Cu, and the low atomic scattering factors of the elements that construct the precipitates. In the Cu-containing alloy aged at 6000 s, many fine precipitates and some short needle-shaped precipitates were observed. The SAED pattern also gives a clear contrast of the extra diffraction spots of the second clusters. At the peak-aged conditions, both of the alloys possess the cross-sectional *β*″ phases (parallel to [001]_α_) and needle-shaped *β*″ phases (parallel to [100]_α_ or [010]_α_) that usually form at the peak hardness. However, the size of the *β*″ phases formed in the Cu-containing alloy is finer due to the effects of the Cu addition [34]. It increases the precipitate density by increasing the rate of cluster nucleation during the initial stage of aging due to the attractive interaction between the Cu and the other solute atoms [35].

Both of the alloys exhibit extra diffractions related to the second clusters at the peak-aged conditions. However, the extra diffraction spots are actually very close to {110}*_β_*_″_ and {511}*_β_*_″_ diffractions from [$\bar{1}$06]*_β_*_″_, which has been reported by Yang et al. [33]. Therefore, it is possible that the diffractions come from both the second clusters and the *β*″ phases. The key diagrams of diffractions from the second clusters and the needle-shaped *β*″ phases obtained when the incident beam and [001]_α_ are parallel to [304]*_β_*_″_ and [$\bar{1}$06]*_β_*_″_ is shown in Figure 4a. The key diagram in Figure 4b displays only the diffractions of the second clusters and the matrix, since some of the second cluster diffractions overlap with the diffractions from [$\bar{1}$06]*_β_*_″_ in Figure 4a. The diffractions from the cross-sectional *β*″ phases are not shown, because they have a relatively weak diffraction effect due to their small size (the diameters are approximately 3~5 nm) and the fact that Al, Mg, and Si have very similar atomic scattering factors [33]. Thus, it is difficult to obtain their diffraction spots with the SAED pattern. On the contrary, due to the large areas of the needle-shaped *β*″ precipitates ([304]*_β_*_″_ and [$\bar{1}$06]*_β_*_″_) when they appear with the longitudinal axis perpendicular to the electron beam direction, they display a much stronger diffraction effect in the SAED patterns. This can also concern the directions of [304]*_β_*_″_ and [$\bar{1}$06]*_β_*_″_, which are high-index zone axes, where the Ewald sphere will cut off both the zero-layer and the ±1 reciprocal planes [33]. Thus, the diffractions from both axes are visible on the [001]_α_ SAED pattern, looking “cross-shaped” [33].

**Figure 5:**
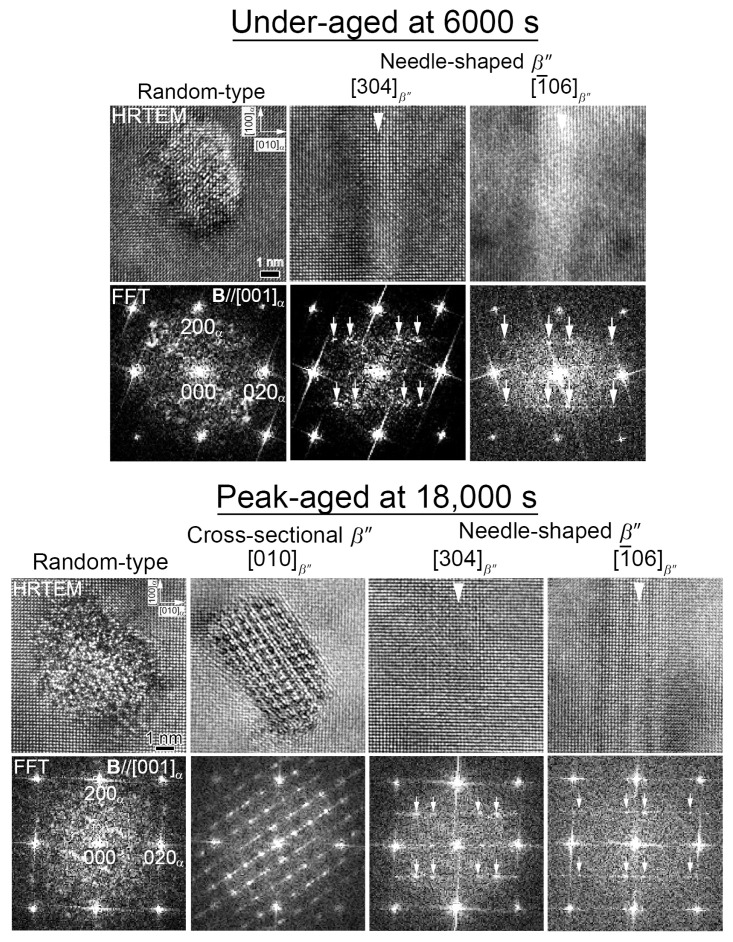
HRTEM images and FFT patterns of precipitates observed in the Cu-free alloy under-aged at 6000 s and peak-aged at 18,000 s.

Figure 5 shows the HRTEM images and their corresponding fast Fourier transform (FFT) patterns of precipitates observed from [001]_α_ of the Cu-free alloy under-aged at 6000 s and peak-aged at 18,000 s. There are random-type precipitates, needle-shaped [304]*_β_*_″_, and [$\bar{1}$06]*_β_*_″_ phases at under-aged conditions. The white arrowheads in the HRTEM images indicate the needle-shaped *β*″ phases, and the white arrows in the FFT patterns indicate their signals corresponding to Figure 4. The types of precipitates observed at the peak-aged conditions are like an under-aged condition with an addition of cross-sectional [010]*_β_*_″_. The atomic arrangement of the precipitates is highly ordered at the peak-aged conditions, except for the random-type precipitates. 

**Figure 6:**
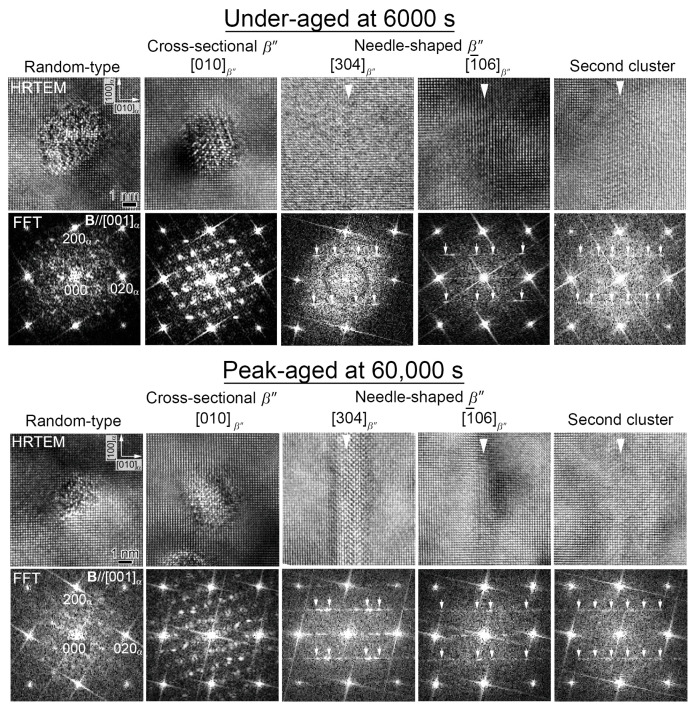
HRTEM images and FFT patterns of precipitates observed in the Cu-containing alloy under-aged at 6000 s and peak-aged at 60,000 s.

Figure 6 shows the HRTEM images and their corresponding FFT patterns of precipitates obtained from [001]_α_ of the Cu-containing alloy under-aged at 6000 s and peak-aged at 60,000 s. In addition to the types of precipitates shown in Figure 5, the needle-shaped precipitates with FFT patterns of the MgSiMg second cluster are additionally observed in both of the conditions, and the needle-shaped precipitates are shorter compared to the *β*″ phases. In addition, this time, the cross-sectional *β*″ phases can be observed in the under-aged conditions because the Cu increases the age-hardening rate. The white arrowheads in the HRTEM images indicate the needle-shaped precipitates, which, in this case, are the [304]*_β_*_″_ and [$\bar{1}$06]*_β_*_″_ zone axes of the *β*″ phases and the second clusters. The presence of the second clusters in the Cu-containing alloy is consistent with that reported by Matsuda et al. [25]. However, such morphology and close observations have never been reported in the Al–Mg–Si alloys. Since the second clusters also have a needle-shaped morphology like the [304]*_β_*_″_ and [$\bar{1}$06]*_β_*_″_ phases and exist as precipitates, the second clusters are referred to as “R phases” here. the volume fractions ($V_{f}$) of the *β*″ and R phases are calculated using Equations (1) and (2) [36,37]. The number density ($N_{d}$) is calculated by dividing the total number of precipitates (n) by the multiplication of the observed area (*A*) and thickness (*t*), which is measured from a convergent beam electron diffraction method. The *A* parameter for each condition is approximately within 300 × 400 to 500 × 600 nm^2^ from the BF images. Two BF images are used to calculate the $N_{d}$ from two areas. The ratio (r) of the *β*″ and R phases is measured by counting their respective frequencies from an average of 69 HRTEM images with an area of 20 × 20 nm^2^ and dividing them by the total frequency of both phases. Therefore, their total r is considered to be one. Using the $N_{d}$ of each phase taken from two different areas and the r of each phase, the average $V_{f}$ is calculated. The $d_{ave}$ and $L_{ave}$ are the average diameter and length of the phases, respectively. Both are measured from the HRTEM images, since the distinction between the *β*″ and R phases is clear using the FFT patterns.

(1) $$N_{d}=\frac{n}{A\times t}$$

(2) $$V_{f}=\frac{\pi d_{ave}^{2}}{4}\times L_{ave}\times r\times N_{d}\times 100$$

**Figure 7:**
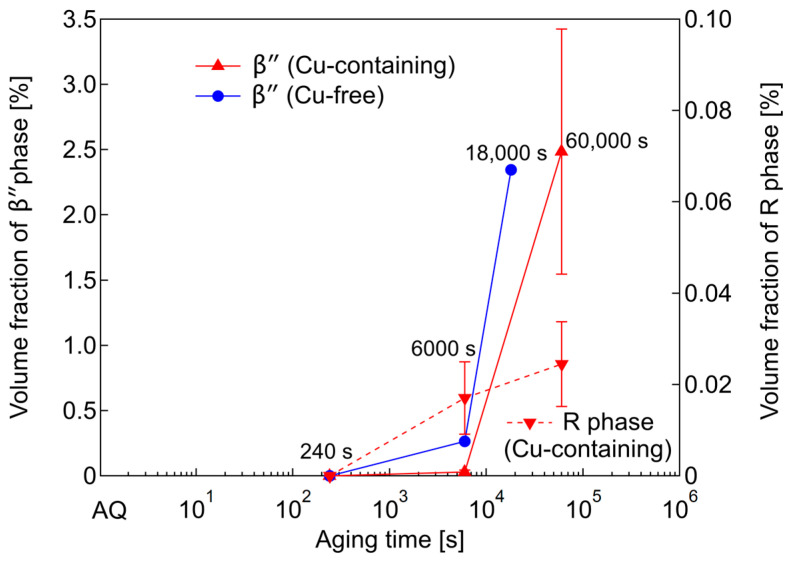
Volume fractions of needle-shaped precipitates observed by TEM at 240- and 6000-s under-aged and peak-aged conditions (18,000 s for Cu-free alloy and 60,000 s for Cu-containing alloy).

Figure 7 shows the volume fraction of the needle-shaped precipitates observed by TEM at 240- and 6000-s under-aged and peak-aged conditions. The volume fractions of the *β*″ phases in both of the alloys and the R phases of the Cu-containing alloy gradually increase with aging. Thus, the R phases also contribute to the hardness. However, in the case of the R phase, while having the same tendency, the volume fraction is relatively small. It is possible that the R phase has low stability.

### 3.3. SANS Analyses

Based on the crystal structures and scattering lengths for the neutrons of Al, Mg, and Si, the differences in the scattering length density between the matrix and the precipitates of the *β*″ ($\Delta \rho_{\beta^{\prime \prime }}$) and R phases ($\Delta \rho_{R}$), where the considered unit cell corresponded to the second cluster, were calculated as 0.522×10^10^ and 0.770 × 10^10^ cm^−2^, respectively.

**Figure 8:**
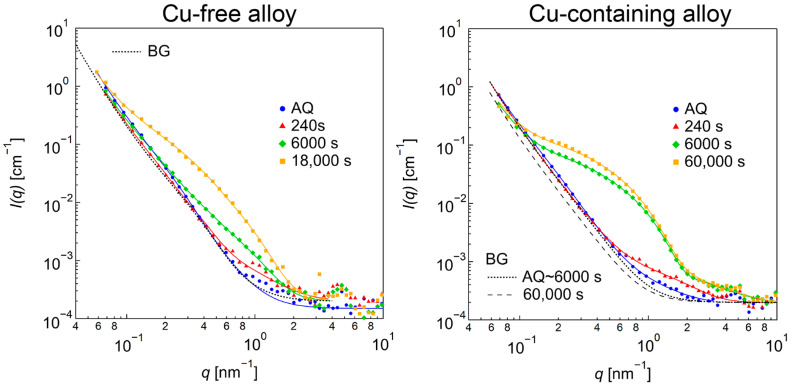
SANS profiles of Cu-free and Cu-containing alloys at AQ, 240, and 6000 s under-aged and peak-aged at 18,000 and 60,000 s, respectively. The dotted and dashed lines show background (BG) caused by microstructures larger than 100 nm. Fitted curves are shown as solid lines.

Figure 8 shows the SANS profiles of the Cu-free and the Cu-containing alloys at AQ, under-aged at 240 and 6000 s and peak-aged at 18,000 s for the Cu-free alloy and 60,000 s for the Cu-containing alloy. The profiles are fitted by the following equation:

(3) $$I\left(q\right)=\Delta \rho_{\beta^{\prime \prime }}^{2}\int V_{\beta^{\prime \prime }}^{2}\left(r\right)F_{\beta^{\prime \prime }}^{2}\left(q,r\right)N_{\beta^{\prime \prime }}\left(r\right)dr+\Delta \rho_{R}^{2}\int V_{R}^{2}\left(r\right)F_{R}^{2}\left(q,r\right)N_{R}\left(r\right)dr+BG,$$

where Δρ is the difference in the scattering length densities between the matrix and the precipitate, $V\left(r\right)$ is the volume of each particle with a size of r, and $N\left(r\right)$ is the volume-weighted size distribution [38]. Here, we used a log-normal distribution for $N\left(r\right)$. $F\left(q,r\right)$ is the form factor depending on the shape of the particle. BG is the background formed by microstructures larger than 100 nm, such as the relatively large compounds formed during solidification. It is determined and fixed as the curve, which can be expressed as the lowest intensity of each q through this aging, which is shown as a dotted curve in Figure 8, except BG for the Cu-containing alloy annealed for 60,000 s, which is shown in the dashed line. This is caused by the change in larger structures, due to the longest aging time.

A form factor that represents an “ellipsoid” of rotation fitting was chosen. The equation can be expressed as follows:

(4) $$F_{ellipsoidal}\left(q,r\right)=\overset{\frac{\pi }{2}}{\underset{0}{\int }}F_{sphere}^{2}\left[q,r\left(a,\frac{c}{a},\alpha \right)\right]sin\alpha \;d\alpha .$$

$$Here\;r\left(a,\frac{c}{a},\alpha \right)=a\sqrt{sin^{2}\alpha +{\left(\frac{c}{a}\right)}^{2}cos^{2}\alpha },$$

where a is the semi-minor axis and c is the semi-major axis [39]. Although the TEM observations showed a needle-like shape for the precipitates, we used an ellipsoidal shape as the reasonable approximation because it can converge much easier and faster than the cylindrical shape.

Compared to the BG curve, there are some extra scatterings observed in the range of 0.8 < q < 3 nm^−1^ for the alloy aged for 240 s corresponding to a single type of nanometer-sized precipitate. Although similar scattering can also be observed even in the AQ, the intensity level is clearly higher in the profile of 240 s, showing that nano-sized precipitates have already formed at this stage in the Cu-free alloy. The corresponding scattering is more prominent in the Cu-containing alloy. These scattering contributions are well explained by the existence of flat particles with a c/a (an aspect ratio of the semi-major axis divided by the semi-minor axis) of less than one. The radii are about 1 and 1.6 nm for the Cu-free and Cu-containing alloys, respectively. In the Cu-free alloy of 6000 s, the intensity of the SANS profile increases in the q range from 0.2 to 2 nm^−1^, indicating the formation of larger particles. Interestingly, the Cu-containing alloy of 6000 s shows clear shoulder-like scattering, indicating that a larger amount of precipitates form compared to that of the Cu-free alloy. Note that a clear kink appears at around 2 nm^−1^ in the profile of the Cu-containing alloy aged for 6000 s, and the q-dependence of the SANS curve is similar with the alloy aged for 240 s in the q-range higher than 2 nm^−1^. This suggests that the single nano-sized particles still exist in this stage. In the peak-aged condition (18,000 s) of the Cu-free alloy, the intensity increases drastically compared to the 6000 s. Although the profile of 18,000 s seems to be attributed to a single-modal ellipsoid, a bimodal size distribution is required to fit the observed curve. On the other hand, the intensity change from 6000 to 60,000 s in the Cu-containing alloys is relatively small, which indicates that the formation kinetics of the large precipitates are quite different between the Cu-free and Cu-containing alloys.

**Figure 9:**
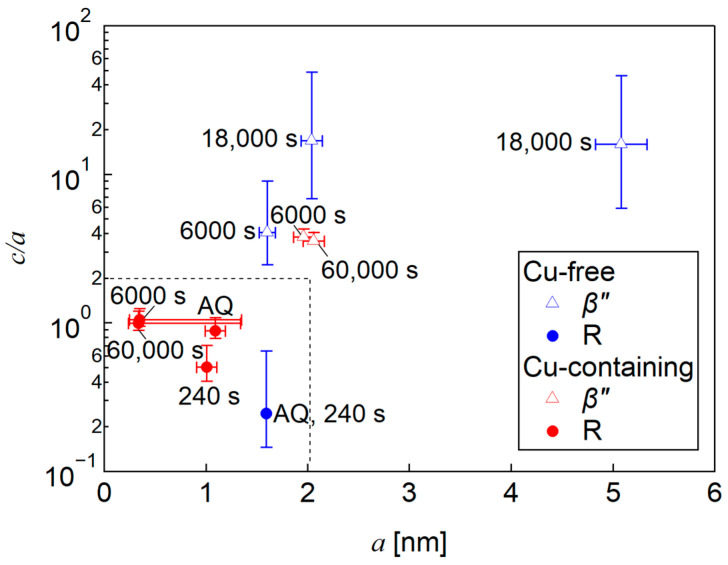
Aspect ratio, c/a vs. semi-minor axis a, map. Open triangles correspond to *β*″ phases, whereas filled circles correspond to R phases in the Cu-free (blue) and Cu-containing alloys (red).

**Figure 10:**
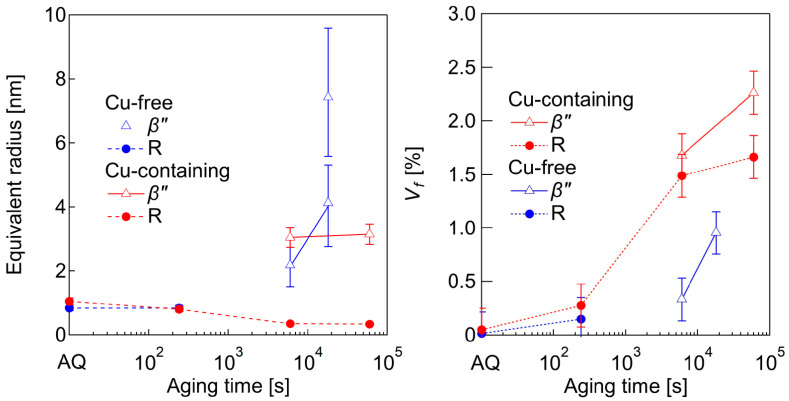
Time evolution of equivalent radius and $V_{f}$ of *β*″ (open triangle) and R phases (filled circle) for the Cu-free and Cu-containing alloys. The Cu-free alloy has two equivalent radii at 18,000 s, resulting from two fittings of *β*″ phases to fit the original curve pattern in Figure 8.

All of the SANS profiles are fitted by using Equations (3) and (4), and the fitted curves are plotted as solid lines in Figure 8. The results of the size evaluation using a form factor of an ellipsoid are plotted as a c/a vs. a map, as shown in Figure 9. By comparing with the TEM observation results, we judged the particles with a c/a below about one to be the R phases (inside the dashed line in Figure 9) and used $\Delta \rho_{R}^{2}$ to evaluate the volume fraction, $V_{f}$, of these particles. For the particles with a c/a larger than two, $\Delta \rho_{\beta^{\prime \prime }}^{2}$ is used to evaluate the $V_{f}$. To see the time evolution of the particles size, an equivalent sphere radius, which has the same particle volume with the ellipsoid shape, is shown in Figure 10 together with the $V_{f}$ for both the R and *β*″ phases. Note that there are two equivalent radii for the Cu-free alloy annealed for 18,000 s due to the bimodal size distribution described above. The smaller particles show a larger $V_{f}$ than the large particles. The $V_{f}$ of 18,000 s in Figure 10 is the sum of both of the particles.

Based on Figure 10, the equivalent radius of the R phases in both of the alloys are constant from AQ to 240 s. After that, the R phases cannot be detected in the Cu-free alloy; furthermore, the R phases might undergo reversion and contribute to the formation of the *β*″ phases. However, in the Cu-containing alloy, the R phases can still be detected. The radius decreases towards 6000 s and remains constant until it is peak-aged. These factors indicate that the R phases grow into an ellipsoidal morphology through in-plane elongation, which simultaneously causes the decrease in its radius. In contrast, the equivalent radius of the *β*″ phases in both of the alloys, which are detected from under-aged 6000 s, is increasing towards peak-aged conditions. Nevertheless, the radius increment in the Cu-containing alloy is relatively small. The reason for this matter is that the Cu segregation behavior on the interfaces hinders radius enlargement and promotes the elongation of the longitudinal axis [14]. Secondly, there is a possibility that the solute atoms are being used not only for the growth of the *β*″ phases, but also for the R phases. Thus, the growth kinetics of the precipitates is small, but the number density is large. 

The $V_{f}$ results show an increase for both of the alloys and both of the precipitates with the aging time. Overall, the $V_{f}$ of both the R and *β*″ phases in the Cu-containing alloy surpasses those of the Cu-free alloy. In addition, this result proves that the R phases contribute to the hardness at the early stage of aging, whereas the *β*″ phases contribute near peak-aging. Then, the increase in $V_{f}$ for the R phases slows down at that stage because the structure becomes unstable with further aging due to the increased strain energy [40]. This gives the *β*″ phases a higher chance to precipitate and grow drastically near to the peak-aged conditions. From Figure 10, we can conclude that the Cu addition somehow promotes the stability of the R phases, and, thus, they remain in the alloy even until peak-aged conditions, unlike in the Cu-free alloy. Additionally, the R phases contribute to the hardness, especially at the early stage of aging.

### 3.4. Unified Interpretations of Random-Type Precipitate and Needle-Shaped R Phase

**Figure 11:**
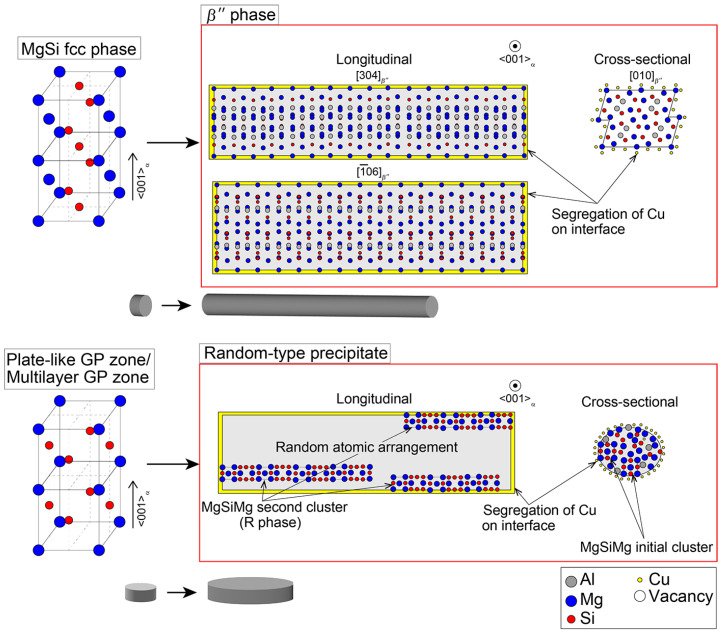
Schematic diagrams of the proposed precipitation behaviors of *β*″ and R phases at 473-K in the Cu-containing alloy.

It is necessary to draw a conclusion that the R phases originated from the random-type precipitates observed along their longitudinal axes, since only two types of needle-shaped precipitates are confirmed in Figure 5, Figure 6 and Figure 8 using both TEM and SANS. This conclusion can also be supported by the previous reports based on the morphology of the random-type precipitates [13]. The formation of precursors prior to the *β*″ and R phases can be distinguished from the thermodynamic simulation results of the Al–Mg–Si alloys, which are carried out using cluster expansion formalism [40]. The parameters are trained by fitting configurational energy data from a density functional theory with a mesoscale phase-field model that includes bulk free energy, surface tension, and strain energy [40]. They proposed two possible precursors, which are a layered MgSi fcc phase and a plate-like GP zone (monolayer GP zone) [3,40]. The layered MgSi fcc phase will phase transform into the strengthening phase *β*″ by forming *β*″-eye in the process [40]. However, it is impossible to observe the layered MgSi fcc phase with HRTEM until a more distinct structure like *β*″-eye is formed [40]. Meanwhile, the plate-like GP zone, which has a stable structure, will grow into a “needle-like GP zone” through in-plane elongation. The plate-like GP zones are the structure that we observed in Figure 2. From the proposed structure of the needle-like GP zone [40], we believe that the needle-like GP zone is the R phase in our research. The strain energy is slightly higher for the needle-like GP zone, indicating that it is less stable compared with the precursor of the *β*″ phase, with further elongation [40]. The proposed precipitation behavior of the *β*″ and R phases in the Cu-containing alloy aged at 473 K is summarized in Figure 11. The schematics shown in Figure 11 indicate the morphologies of each precipitation phase, which are shown in gray.

Based on all of these results, it is suggested that the R phases do form in both the Cu-free and the Cu-containing alloys, even though we confirm the presence of “a part of” the R phases in the HRTEM images and FFT patterns of the Cu-free alloy (Figure 5) as a random-type precipitates. The neutron scatterings enable us to detect the R phases because of the different large scattering abilities for the constitutive elements. However, at the present stage, it is hard to differentiate the crystal structure of the R phases confirmed in the Cu-free alloy. There are three reasons for the absence of R phases in the TEM results of the Cu-free alloy. Firstly, R phases could exist in the random-type precipitates that are low-ordered structure precipitates. Secondly, the Al, Mg, and Si atomic numbers are close, resulting in low scattering effects. Lastly, the Cu segregations decrease the strain energy of the R phases, making them more stable to grow in the Cu-containing alloy. The crystal structure of the R phases could stochastically be random when it is cut perpendicular to the longitudinal direction, as shown in Figure 5, Figure 6 and Figure 11. However, the domains with unit cells of the second cluster are partially formed in some areas within the needle-shaped morphology. This is the reason why we show the two different crystal structures of “random” and “R” in the HRTEM images, although they could be consistent. 

## 4. Conclusions

Nanostructural investigations of pseudo-binary Al–1.0Mg_2_Si (mass%) alloys with and without 0.5Cu have been conducted by using TEM and SANS. Both of the alloys exhibit extra electron diffractions related to MgSiMg second clusters in their SAED patterns at peak-aged conditions. The second cluster diffractions are also confirmed from the HRTEM images and FFT patterns of the needle-shaped precipitates in the Cu-containing alloy. The needle-shaped *β*″ phases also precipitate simultaneously with the second clusters. The second clusters are referred to as R phases so that they are related to the so-called “random-type precipitates.” The HRTEM images show that the R phases have shorter and thicker needle-shaped morphologies compared to the *β*″ phases. The R phases are very difficult to observe with TEM, which suggests that they have a low-order structure along its longitudinal axis. We found that the R phases are more likely to form partially along the longitudinal axis of the random-type precipitates, unlike the previous interpretations. Thus, the atomic arrangement in the random-type precipitates is not completely random. Therefore, the changes in the size and volume fraction of the R phases can only be accurately analyzed with SANS. The fitting of the SANS profiles confirms the presence of the *β*″ and R phases in both the Cu-free and the Cu-containing alloys. Evidently, the R phases can form even in the absence of Cu, although we could not confirm their presence by HRTEM. However, according to the SANS results, the R phases cannot be detected after 240 s in the Cu-free alloy. Meanwhile, for the Cu-containing alloy, the presence is confirmed until it is peak-aged, with a drastic increment from 240 s to 6000 s. Surprisingly, the volume fraction of both the R and the *β*″ phases in the Cu-containing alloy surpasses that of the Cu-free alloy. It is clear that the R phases also contribute to the increase in hardness in the Cu-containing alloy. The Cu addition has the effect of stabilizing the growth of the R phases by decreasing their strain energy, in addition to promoting more nucleation at the early stage of aging.

## Figures and Tables

**Table 1:** Chemical compositions of the investigated alloys.

	Alloys	Mg	Si	Cu	Al
mass%	Cu-free	0.63	0.37	0	bal.
	Cu-containing	0.63	0.37	0.50	bal.
mol%	Cu-free	0.70	0.36	0	bal.
	Cu-containing	0.70	0.36	0.21	bal.

## Data Availability

Data will be made available upon request.
